# Supplementary material for: R-spondins are BMP receptor antagonists in Xenopus early embryonic development
Source: Nat Commun. 2020 Nov 4;11:5570. doi: 10.1038/s41467-020-19373-w (PMC7642414; doi:10.1038/s41467-020-19373-w)
Supplement: Supplementary file 1 — Supplementary Information [file 41467_2020_19373_MOESM1_ESM.pdf]

Supplementary Information

R-spondins are BMP receptor antagonists in *Xenopus* early embryonic development

Hyeyoon Lee, Carina Seidl, Rui Sun, Andrei Glinka and Christof Niehrs

Supplementary Figures 1-11

Supplementary Tables 1-4

Supplementary Methods

Supplementary Fig. 1

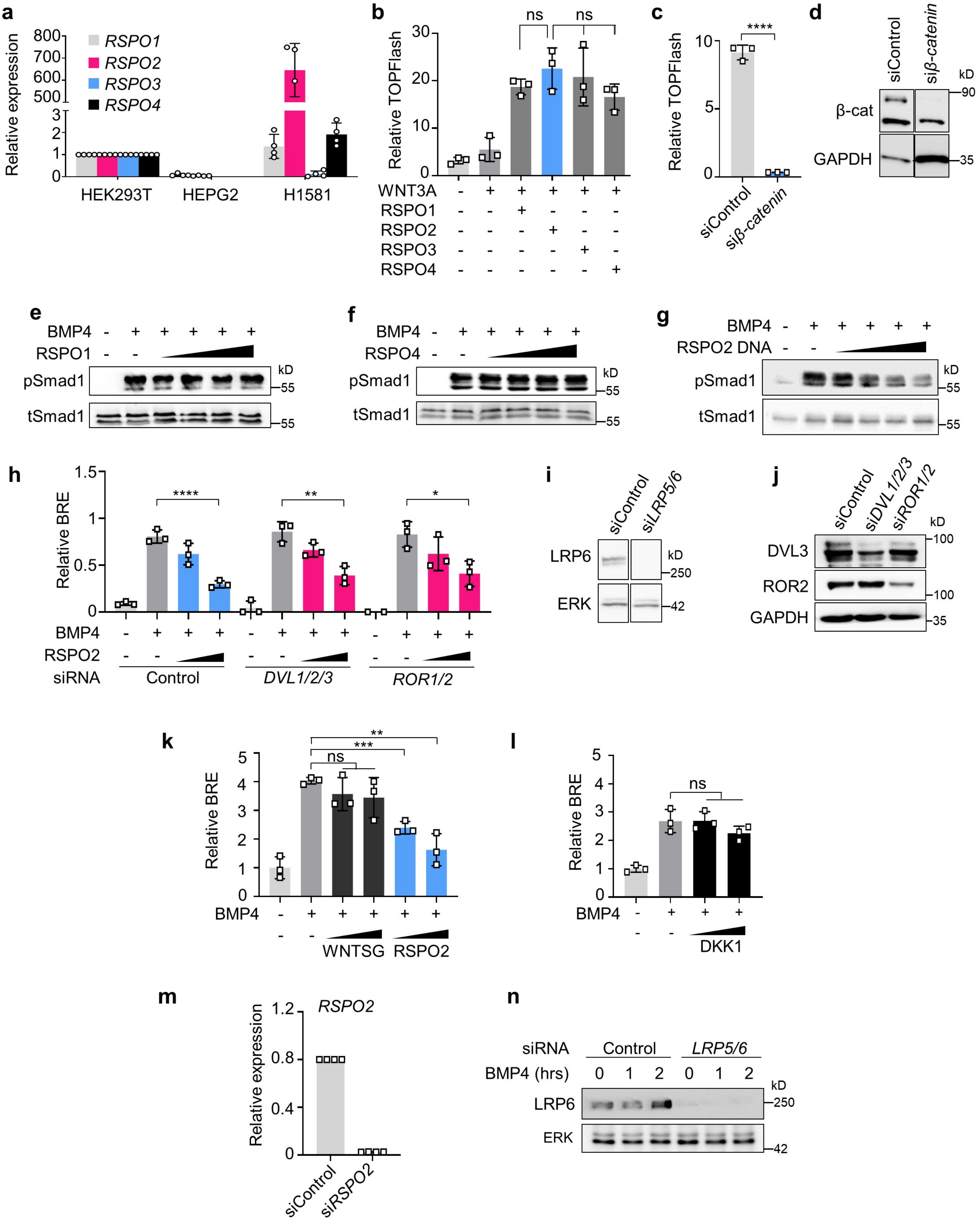

Supplementary Fig. 1. Validation of RSPOs and siRNAs.

(**a**) qRT-PCR analysis of *RSPO1-4* in HEK293T, HEPG2 and H1581 cells. Gene expression in HEK293T cells was set to 1. Data are displayed as means ± SD. n=3-4 experimentally independent samples.

(**b**) TOPFlash assay in HEK293T cells upon overnight treatment with WNT3A with or without RSPO1-4 as indicated. n=3 biologically independent samples.

(**c**) TOPFlash assay in HEPG2 cells treated with siControl or si*β-catenin*, validating knockdown effect of *β-catenin* on WNT signaling, related to **Fig. 1b.** n=3 biologically independent samples.

(**d**) Western blot analyses of β-catenin in HEPG2 cells treated with indicated siRNAs. GAPDH, loading control, related to **Fig. 1b**.

(e-g) Western blot analyses of phosphorylated- (pSmad1) and total Smad1 (tSmad1) in HEPG2 cells upon treatment with BMP4, with or without overnight RSPO1 (**e**) or RSPO4 (**f**) treatment, or RSPO2 DNA transfection (**g**). Data shows representative result from 2 independent experiments with similar conclusion.

(**h**) BRE reporter assay in HEPG2 cells upon siRNA transfection, with or without overnight BMP4 and RSPO2 treatment. n=3 biologically independent samples.

(**i-j**) Western blot analysis in HEPG2 cells upon siRNA transfection to validate knockdown efficiency, related to **Fig. 1g (i)** and **Supplementary Fig. 1h (j)**.

(**k-l**) BRE reporter assays in HEPG2 cells overnight treated as indicated. WNTSG, Wnt surrogate. n=3 biologically independent samples.

Data for reporter assays (**b, c, h, k, l**) are displayed as means ± SD. ns, not significant; $*P < 0.05$ $**P < 0.01$, $***P < 0.001$ from two-tailed unpaired t-test (**c, k, l**) or one-way ANOVA with Dunnett's test (**b, h**).

(**m**) qRT-PCR analysis of *RSPO2* in H1581 cells treated with siControl or si*RSPO2* to validate knockdown efficiency, related to **Fig. 1k**. n=4 experimentally independent samples. Data are displayed as means ± SD.

(**n**) Western blot analysis in H1581 cells treated with siRNA and BMP4 as indicated, related to **Fig. 1l**. Data shows representative result from 2 independent experiments with similar conclusion.

Supplementary Fig. 2

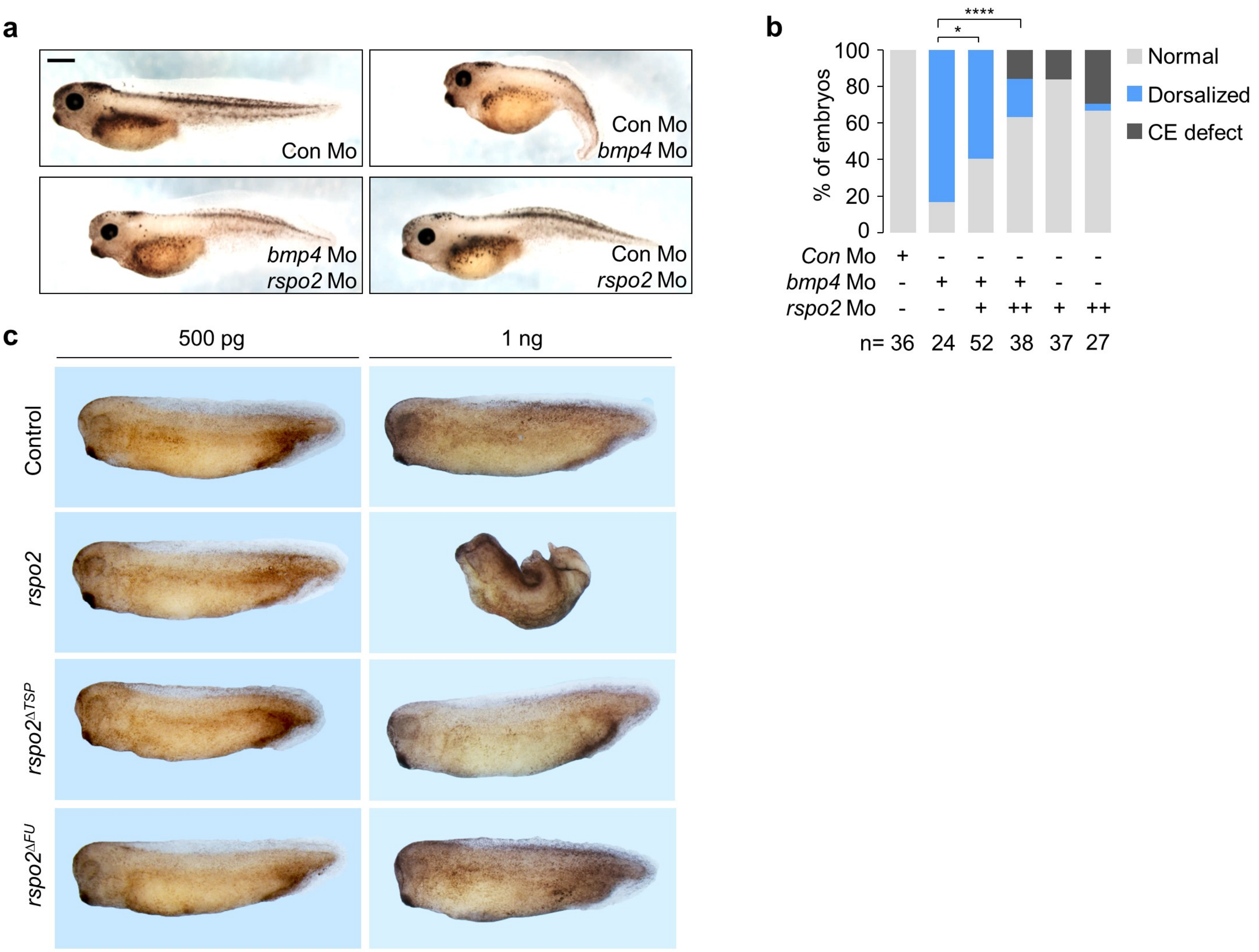

Supplementary Fig. 2. Rspo2 and -3 can inhibit BMP signaling in *Xenopus* embryogenesis.

(**a**) Representative phenotypes of *Xenopus laevis* tadpoles radially injected at 4-cell stage with the indicated Mos. 'Dorsalized' represents bent and reduced ventral structure with slightly enlarged head, similar to *bmp4* Mo phenotypes. Scale bar, 0.5 mm.

(**b**) Quantification of embryonic phenotypes shown in (**a**). CE, convergent extension (gastrulation defects). n=the number of embryos. Data are pooled from three independent experiments. $^{*}P < 0.05$, $^{****}P < 0.0001$ from two-tailed $\chi 2$ test comparing normal versus dorsalized.

(**c**) Representative phenotypes of *Xenopus laevis* tadpoles injected with 500 pg or 1 ng of the indicated *rspo2* mRNA per embryo. Note that only high (1 ng) *rspo2* wildtype mRNA induced gastrulation defects.

Supplementary Fig. 3

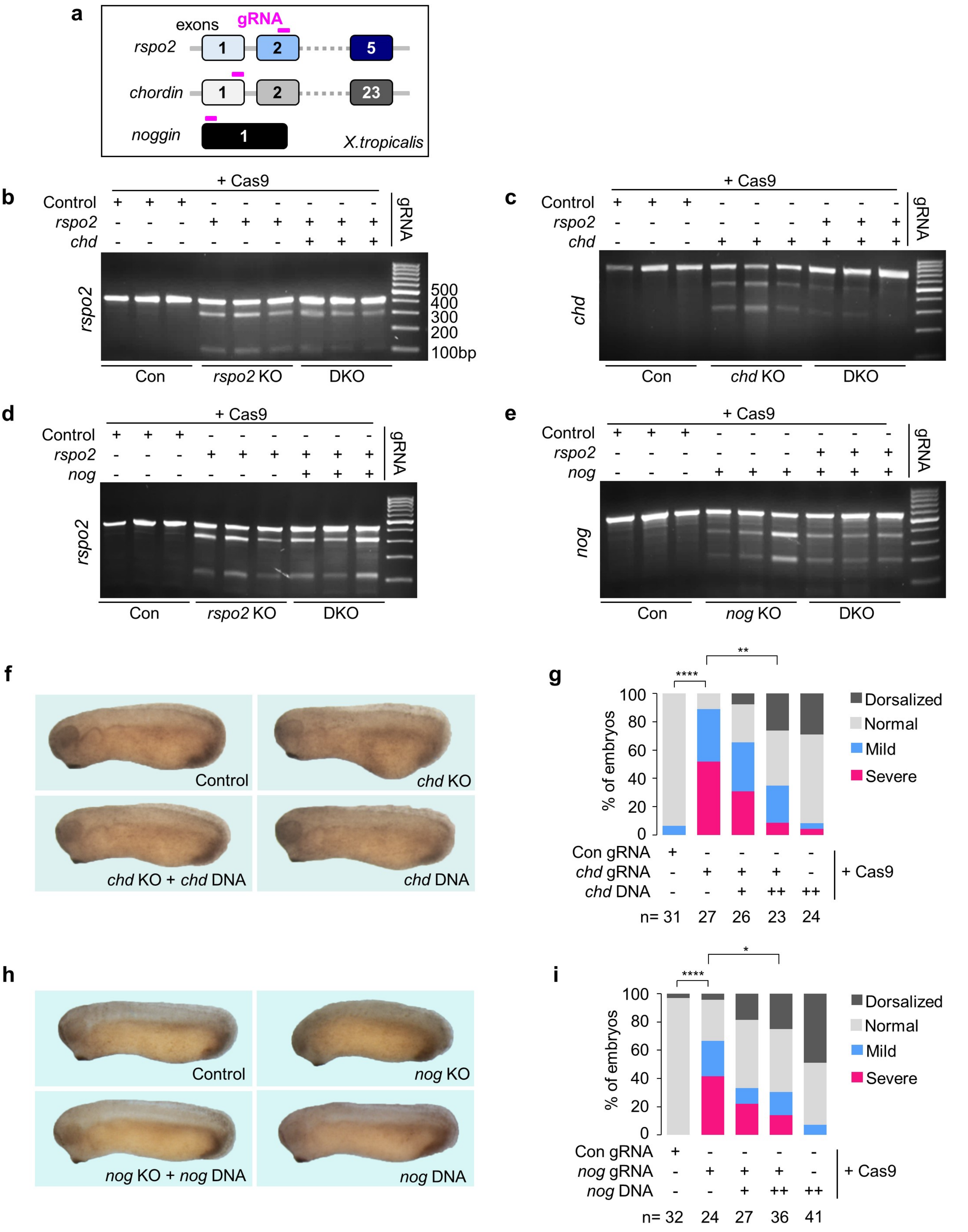

Supplementary Fig. 3. Validation of *rspo2, chordin* and *noggin* guide RNAs in *Xenopus tropicalis.*

(**a**) Schemes for guide RNA (gRNAs) targeting rspo2, chordin, and noggin in Xenopus tropicalis embryos. gRNAs are indicated with red bar on top of targeting sites. Exons are indicated as boxes with numbers.

(**b-e**) Agarose gel electrophoresis of amplified and T7E1 incubated genes from St. 30 embryos injected with gRNAs and Cas9 proteins as indicated at 1-cell stage. rspo2, chordin or noggin gDNAs were PCR amplified. After T7E1 incubation, Cas9-mediated genome editing yields DNA fragments. DKO, double knockout of rspo2/noggin or rspo2/chordin. Data show 3 representative Crispants per set. The experiment was performed 3 times.

(**f, h**) Representative phenotypes of *Xenopus tropicalis* tadpole Crispants co-injected with chd (f) or nog (h) DNA at 1 cell stage as indicated.

(**g, i**) Quantification of embryonic phenotypes shown in (**f, h**). 'Severe' represents two or three of defects among small head, enlarged ventral tissues and short body axis. 'Mild' represents one of the defects described above. 'Normal' represents no visible differences to the uninjected control. n= number of embryos. For (**g, i**), *P < 0.05, **P < 0.01, ***P < 0.001, ****P<0.0001 from two-tailed $\chi 2$ test comparing normal versus severe and mild defects.

Supplementary Fig. 4

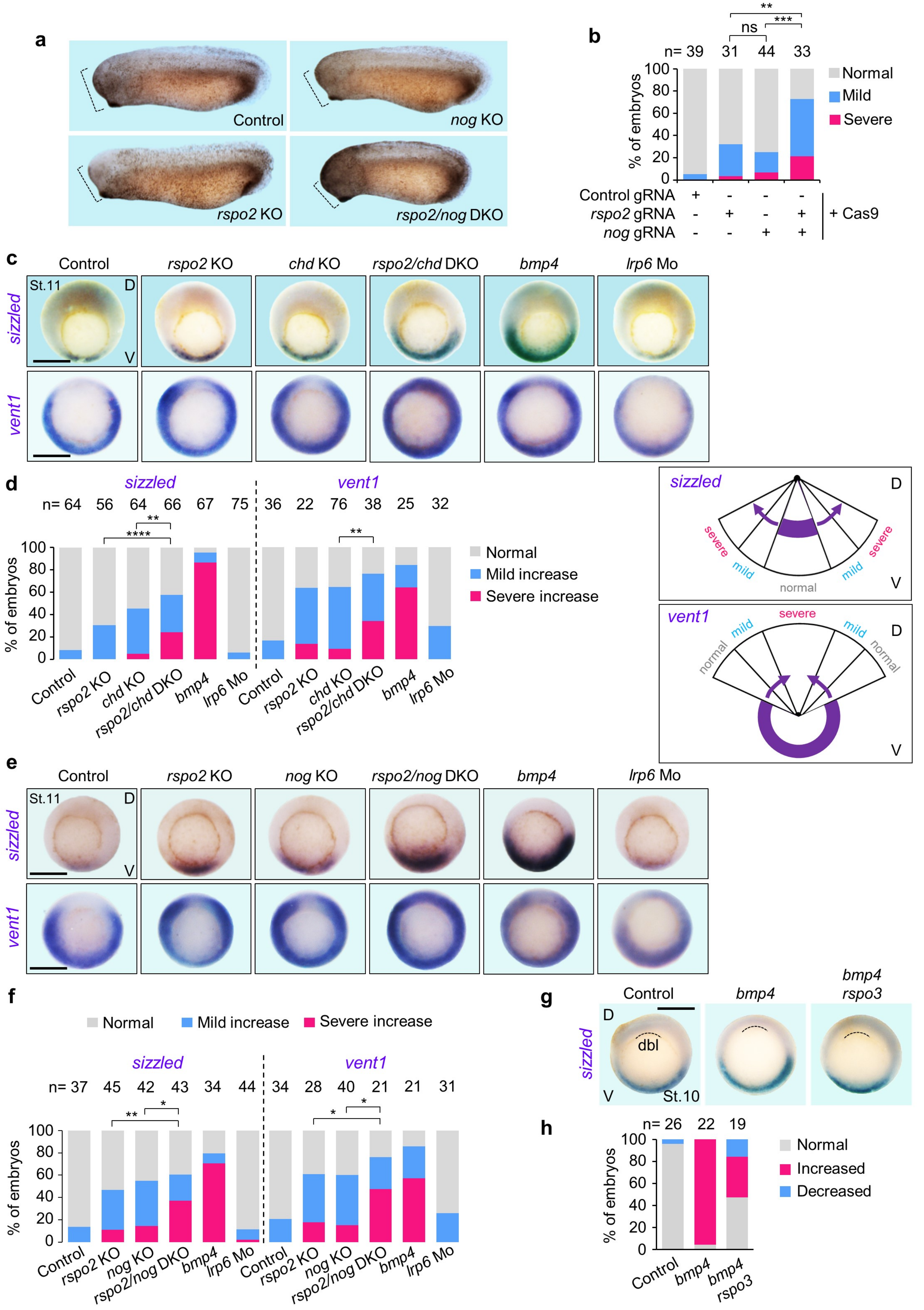

Supplementary Fig. 4. Double knockout of *Xenopus rspo2* and BMP antagonists hyperactivates BMP signaling.

(**a**) Representative phenotypes of *Xenopus tropicalis* tadpole Crispants as indicated. Dashed lines, head size. 'Severe' represents small head, enlarged ventral tissues and short body axis. 'Mild' represents one or two of the defects described above. 'Normal' represents no visible differences to the uninjected control.

(**b**) Quantification of embryonic phenotypes shown in (**a**). Soring of phenotypes was executed blind. 'Severe' represents small head, enlarged ventral tissues and short body axis. 'Mild' represents one or two of the defects described above. n= number of embryos. ns, not significant. $**P < 0.01$, $***P < 0.001$ from two-tailed $\chi 2$ test comparing normal versus severe and mild defects.

(**c**) *In situ* hybridization of *sizzled* and *vent1* in *Xenopus tropicalis* gastrula (St.11, dorsal to the top, vegetal view) Crispants or embryos injected as indicated. D, dorsal, V, ventral. Scale bar, 0.5 mm.

(**d**) (Left) Quantification of phenotypes shown in (**c**). (Right) Scheme for quantification. Purple arc denotes expression of *sizzled* or *vent1*. 'Mild' and 'Severe' embryos were categorized based on how severely *sizzled* or *vent1* signal was expanded dorsally, by measuring the angle of expression. n=the number of biologically independent embryos.

(**e**) *In situ* hybridization of BMP4 targets *sizzled* and *vent1* in *Xenopus tropicalis* gastrula (St.11, dorsal to the top, vegetal view) Crispants or embryos injected as indicated. D, dorsal, V, ventral. Scale bar, 0.5 mm.

(**f**) Quantification of phenotypes shown in (**e**). n= number of embryos.

For (**d, f**), $*P<0.5$, $**P < 0.01$, $***P < 0.001$, $****P<0.0001$ from two-tailed $\chi 2$ test comparing normal and mild versus severe to analyze increase of severity. Data from (**d, f**) are pooled from two independent experiments.

(**g**) *In situ* hybridization of *sizzled* in *Xenopus laevis* gastrulae (St.10, dorsal to the top, vegetal view) injected with *bmp4* and *rspo3* mRNA radially at 4-cell stage. Dashed lines, dorsal blastopore lip (dbl).

(**h**) Quantification of embryonic phenotypes shown in (**g**). 'Increased' represents expansion of *sizzled* expression to the dorsal side compared to control embryos. 'Decreased' represents reduction of the expression. n= number of embryos.

Supplementary Fig. 5

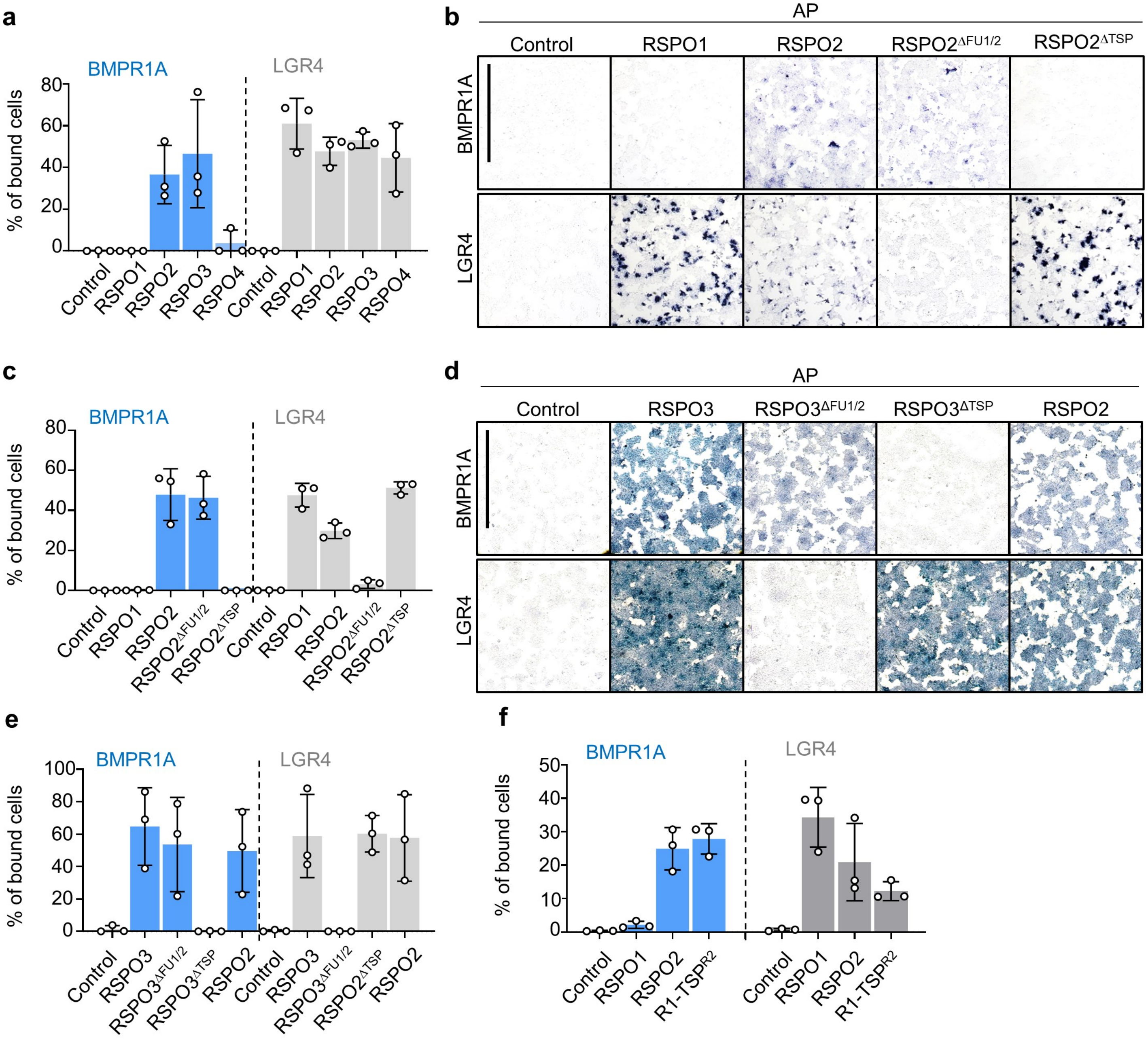

Supplementary Fig. 5. RSPO2 and -3 require the TSP1 domain for BMPR1A binding.

(**a**) Quantification of cell surface binding assay in **Fig. 4d**. n=3 biologically independent samples. Data are displayed as means ± SD.

(**b**) Cell surface binding assay in HEK293T cells for RSPO2 deletion mutants with BMPR1A and LGR4 interaction. Cells were transfected with BMPR1A or LGR4 DNAs, and treated with the same units of RSPO1, RSPO2 or RSPO2 deletion mutants-AP fusion protein after DSP crosslinking. Binding was detected as dark purple cell surface stain by chromogenic AP assay. Data shows a representative from three independent experiments. Scale bar, 1 mm.

(**c**) Quantification of (**b**). n=3 biologically independent samples. Data are displayed as means ± SD.

(**d**) Cell surface binding assay in HEK293T cells for RSPO3 deletion mutants with BMPR1A and LGR4 interaction. Data shows a representative from three independent experiments. Scale bar, 1mm.

(**e**) Quantification of (**d**). n=3 biologically independent samples. Data are displayed as means ± SD.

(**f**) Quantification of cell surface binding assay in HEK293T cells for RSPO1, -2 and R1-TSP^{R2} with BMPR1A and LGR4 interaction. n=3 biologically independent samples. Data are displayed as means ± SD.

Supplementary Fig. 6

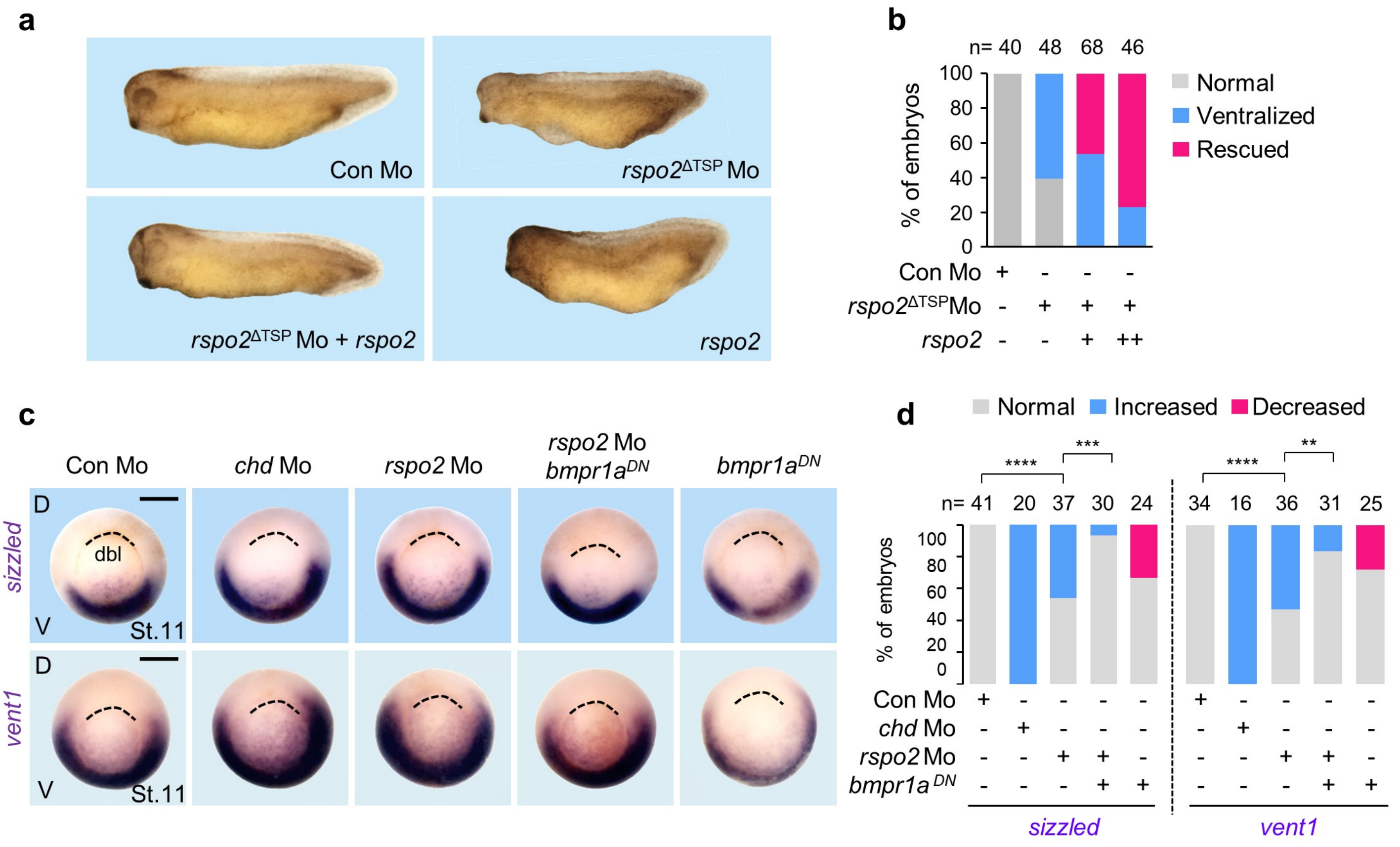

Supplementary Fig. 6. Loss of *Xenopus rspo2* increases BMP signaling.

(**a**) Representative phenotypes of *Xenopus laevis* tadpoles radially injected at 4-cell stage with the Mo and mRNA indicated. (**b**) Quantification of (**a**). ‘Ventralized’ represents small head, enlarged ventral tissues and short body axis. ‘Rescued’ represents one or two of the defects described above, or same as control embryos. n= number of embryos.

(**c**) *In situ* hybridization of BMP target gene *sizzled* and *vent1* in *Xenopus laevis* gastrulae (St.11) radially injected at 4-cell stage as indicated. Dashed line, dorsal blastopore lip (dbl). D, dorsal; V, ventral. Scale bar, 0.5 mm.

(**d**) Quantification of phenotypes shown in (**c**). ‘Increased’ represents embryos with expansion of *sizzled* or *vent1* signals towards the dorsal side of the embryo. ‘Decreased’ represents embryos with decrease of the signal area or the signal strength. ns, not significant. $**P < 0.01$, $***P < 0.001$, $****P<0.0001$ from two-tailed $\chi 2$ test comparing normal versus increased expression. n=the number of embryos. Data are pooled from two independent experiments.

Supplementary Fig. 7

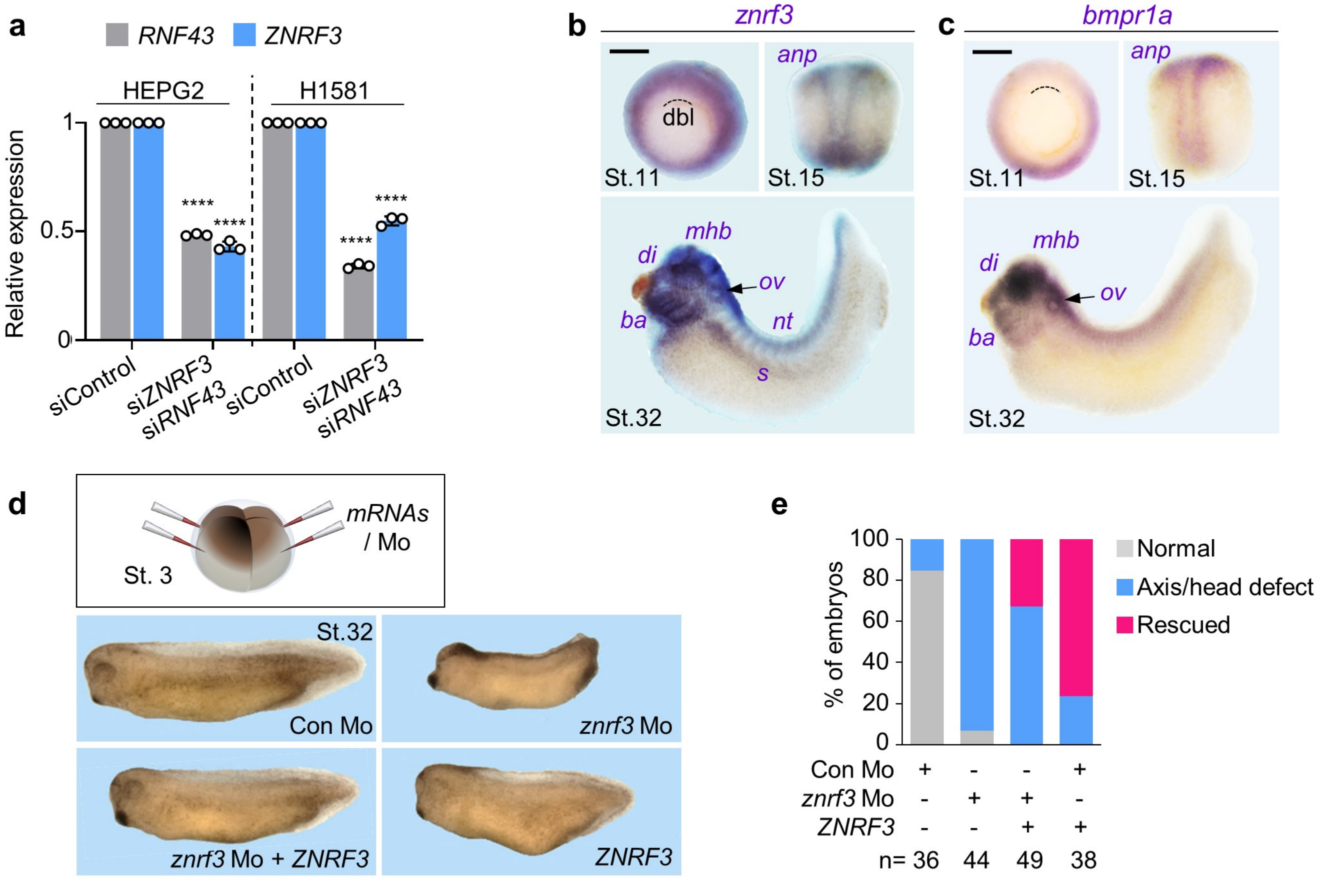

Supplementary Fig. 7. Expression and knockdown of *ZNRF3* in *Xenopus* embryos and culture cells.

(**a**) qRT-PCR analyses of *RNF43* and *ZNRF3* in HEPG2 and H1581 cells treated with indicated siRNA. Expression of *RNF43* and *ZNRF3* in siControl treated cells was set to 1. n=3 experimentally independent samples and data are displayed as mean ± SD. ****P<0.0001 from two-tailed unpaired t-test.

(**b-c**) *In situ* hybridization of *znrf3* (**b**) and *bmpr1a* (**c**) in *Xenopus laevis* at at gastrula (St. 11, dorsal to the top, vegetal view), neurula (St. 15, anterior to the top, dorsal view), and tadpole (St. 32, anterior to the left, lateral view). Dashed line, dorsal blastopore lip (dbl). anp, anterior neural plate. di, diencephalon. mhb, mid-hindbrain boundary. ov, otic vesicle. ba, branchial arches. nt, neural tube. s, somite. Data shows representative images from 3 independent experiments with similar expression patterns.

(**d**) Microinjection strategy and representative phenotypes of *Xenopus laevis* tadpoles injected with the indicated Mo and mRNA radially at 4-cell stage.

(**e**) Quantification of embryonic phenotypes shown in (**d**). 'Axis and head defect' represents defects related to BMP as well as WNT/LRP and WNT/PCP signaling. 'Rescued' represents tadpoles restored from bent axis or small head size. Data are pooled from 3 independent experiments. n= number of embryos.

Supplementary Fig. 8

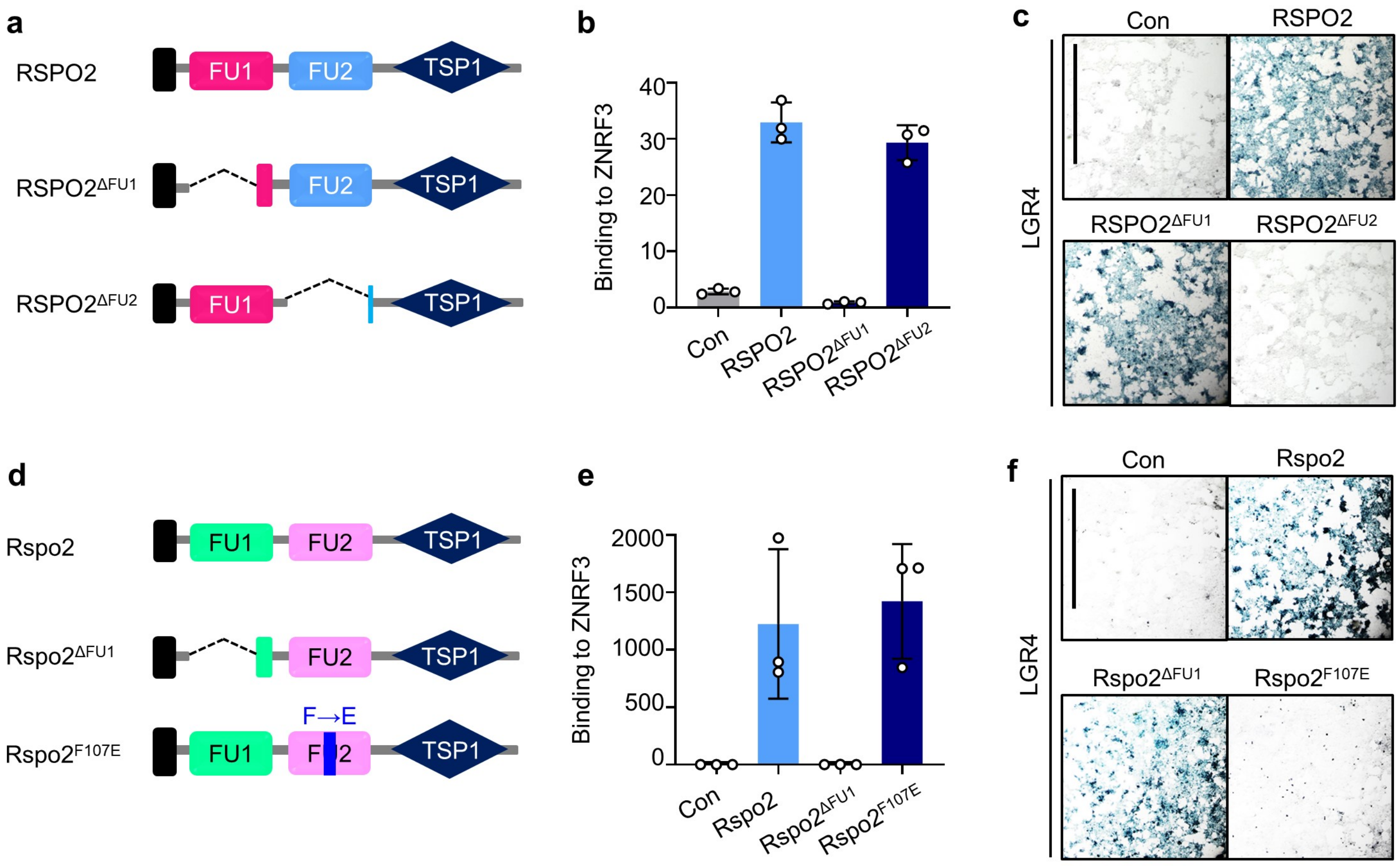

Supplementary Fig. 8. RSPO2 FU1- and TSP1 domain are required for ZNRF3-RSPO2-BMPR1A complex formation.

(**a**) Domain structure of human RSPO2 deletion mutants used for (**b, c**) and **Fig. 7f-g.**

(**b**) *In vitro* binding assay between ZNRF3 and RSPO2 deletion mutants. Immobilized ZNRF3-Fc protein was used as a bait and incubated with RSPO2 or RSPO2 deletion mutant-AP fusion protein overnight. RSPO2 bound to ZNRF3 was detected by chromogenic AP assay. Note that $RSPO2^{\Delta FU1}$, but not $RSPO2^{\Delta FU2}$, lost ZNRF3 binding, validating deletion of the FU1 domain. n=3 experimentally independent samples. Data are displayed as means ± SD.

(**c**) Cell surface binding assay between LGR4 and RSPO2 deletion mutants. Note that $RSPO2^{\Delta FU2}$, but not $RSPO2^{\Delta FU1}$, lost LGR4 binding, validating deletion of the FU2 domain. Data shows images representative for 3 from 5 experimentally independent experiments. Scale bar, 1 mm.

(**d**) Domain structures of *Xenopus laevis* Rspo2 mutants used for **Fig. 7g-h**.

(**e**) *In vitro* binding assay between ZNRF3 and *Xenopus* Rspo2 mutants. Note that $Rspo2^{\Delta FU1}$, but not $Rspo2^{F107E}$, lost ZNRF3 binding, validating deletion of the FU1 domain. n=3 experimentally independent samples. Data are displayed as means ± SD.

(**f**) Cell surface binding assay between LGR4 and *Xenopus* Rspo2 mutants. Note that $Rspo2^{F107E}$, but not $Rspo2^{\Delta FU1}$, lost LGR4 binding, validating mutation of the FU2 domain. Data shows representative images from 2 independent experiments with similar conclusion. Scale bar, 1 mm.

Supplementary Fig. 9

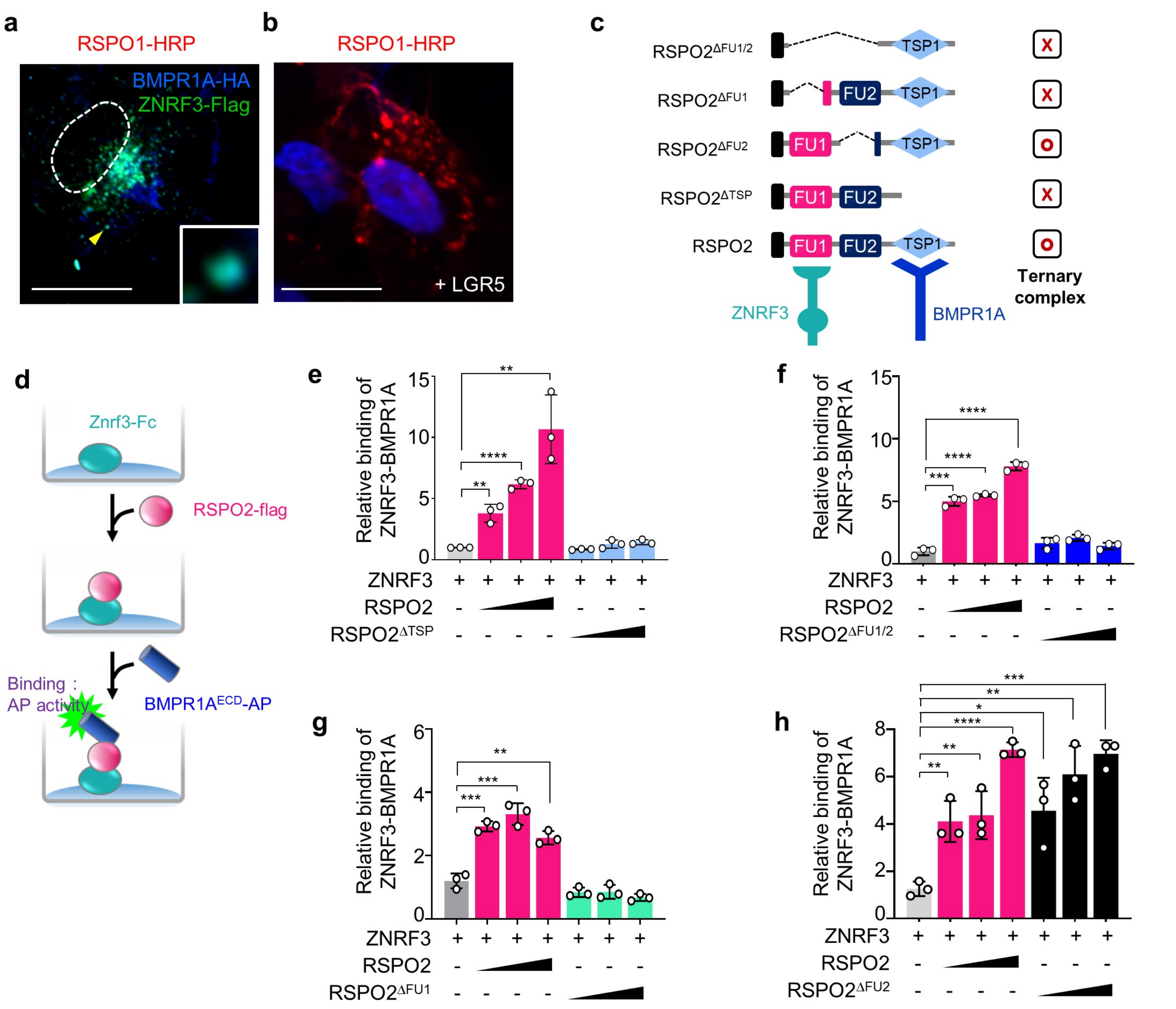

Supplementary Fig. 9. RSPO2-TSP1 and FU1 domains are required for ZNRF3-RSPO2-BMPR1A complex formation.

(**a, b**) Immunofluorescence microscopy (IF) in H1581 cells transfected with BMPR1A-HA and ZNRF3-flag DNA (**a**) or LGR5 DNA (**b**) upon 3 hours RSPO1-HRP treatment. RSPOs (red) were visualized by tyramid signal amplification. BMPR1A (blue) and ZNRF3 (green) were stained by HA and Flag antibody (**a**) and nuclei were stained with Hoechst (**b**). Note that RSPO1 was not internalized with BMPR1A and ZNRF3 and hence was undetectable, whereas LGR5 mediates RSPO1 internalization, confirming RSPO1-HRP activity. Yellow arrowhead, colocalized BMPR1A/ZNRF3 in magnified inset. Data shows representative images from 8-20 individual cells with similar conclusion. Scale bar, 20 μm.

(**c**) Domain structure of RSPO2 deletion mutants and summary for ZNRF3-RSPO2-BMPR1A^{ECD} binding assay in (**e-h**).

(**d**) Scheme for ZNRF3-RSPO2-BMPR1A^{ECD} binding assay. ZNRF3-Fc protein was used as a bait, with sequential addition of RSPO2/RSPO2 deletion mutant-flag conditioned media and BMPR1A^{ECD}-AP. BMPR1A^{ECD}-AP bound to ZNRF3 was detected by chromogenic AP assay.

(**e-h**) *In vitro* binding assay between ZNRF3 and BMPR1A^{ECD} mediated by RSPO2 wild type and deletion mutants. n=3 experimentally independent samples. Data are displayed as means ± SD. **P < 0.01, ***P < 0.001, ****P<0.0001 from two-tailed unpaired t-test.

Supplementary Fig. 10

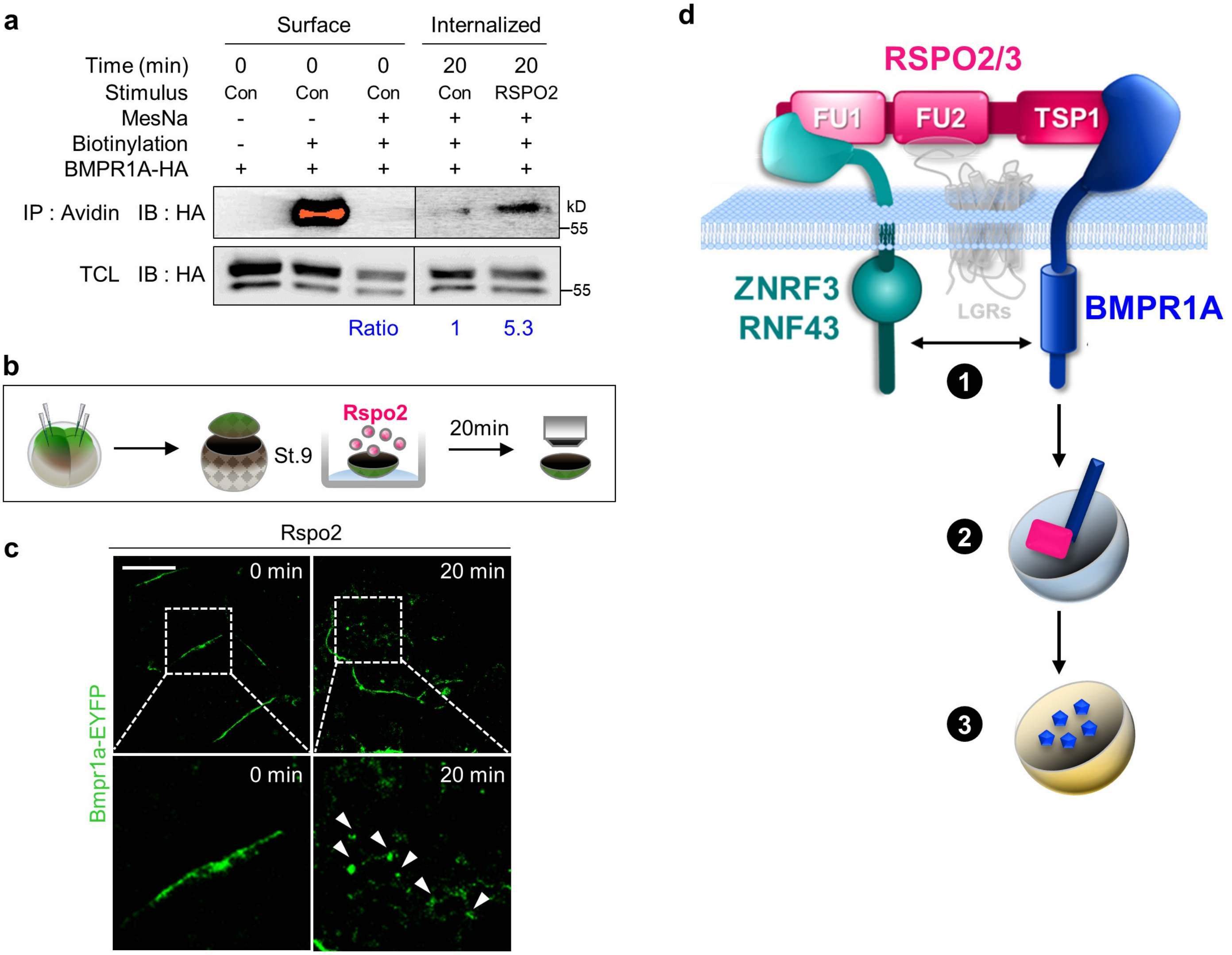

Supplementary Fig. 10. RSPO2 mediates BMPR1A internalization.

(**a**) Receptor internalization assay in H1581 cells following BMPR1A-HA transfection and treatment with control medium (Co) or RSPO2 as indicated. After labeling surface proteins with cleavable biotin on ice, cells were stimulated with control medium or RSPO2 for 20 min at 37 °C to induce internalization. MesNa (2-Mercaptoethansulfonat-Natrium) was added to remove biotin from remaining surface proteins. Lysates were pulled down with streptavidin beads and subjected to Western blot analysis. TCL, Total cell lysate. Ratio, internalized BMPR1A-HA levels normalized to BMPR1A-HA. Data shows representative results from 2 independent experiments with similar conclusion.

(**b**) Scheme for immunofluorescence microscopy (IF) of *Xenopus laevis* animal cap explants shown in (**c**) bmpr1a-EYFP mRNA was injected animally in 4-cell stage embryos and animal cap explants were dissected at stage 9. *Xenopus Rspo2* conditioned medium was added for 20 min and animal cap explants were analyzed by IF.

(**c**) IF of animal cap explants (see b) for Bmpr1a. Shown is one representative cell (top) and magnification (inset). Arrowheads indicate internalized Bmpr1a-EYFP punctae. Data shows representative images from 10 biologically independent animal cap tissues with similar results. Scale bar, 20 µm.

(**d**) Model showing (1) ternary complex of RSPO2-ZNRF3-BMPR1A mediating (2) membrane clearance and (3) degradation of BMPR1A.

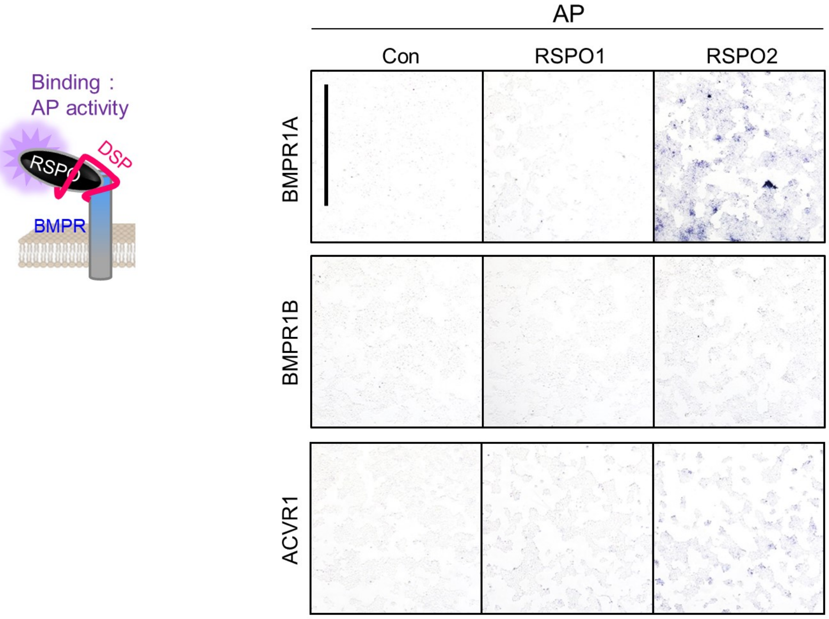

Supplementary Fig. 11. RSPO2 specifically binds to BMPR1A.

Cell surface binding assay in HEK293T cells. (Left) Scheme of the assay. Cells were transfected with BMPR1A, BMPR1B or ACVR1 DNA, and treated with same amount of RSPO1-2-AP upon DSP crosslinking as indicated. Binding was detected as purple stain on cell surface by chromogenic AP assay. (Right) Images of cells transfected and treated as indicated. Data shows a representative from 1-3 biologically independent experiments show similar results. Scale bar, 1 mm.

Supplementary Tables

	Gene	Forward (F) and Reverse (R) primers
Human	*RSPO1*	F: ATCAAGGGGAAAAGGCAGA
		R: CAGAGCTCACAGCCTTTGG
	RSPO2	F: TGTCCAACCATTGCTGAATC
		R: TCCTCTTCTCCTTCGCCTTT
	RSPO3	F: AAGTGTCAGAAGGGAGAACGAG
		R: TGCTGTCAGGTATTGCTTCTTT
	RSPO4	F: TTTGGCCCACCAGAACAC
		R: CCGCAGGTCTTTCCATTG
	ID1	F: CCAGAACCGCAAGGTGAG
		R: GGTCCCTGATGTAGTCGATGA
	ZNRF3	F: TGTGCCATCTGTCTGGAGAA
		R: TTCCTGTGAAACCGGTGAGT
	RNF43	F: GTTTGCTGGTGTTGCTGAAA
		R: TGGCATTGCACAGGTACAG
	GAPDH	F: AGCCACATCGCTCAGACAC
		R: GCCCAATACGACCAAATCC
X.laevis	*rspo2*	F: CCAGCTATGGGACCAATCC
		R: CGGAGGCACCCATTATCTT
	vent1	F: GGCACCTGAACGGAAGAA
		R: GATTTTGGAACCAGGTTTTGAC
	sizzled	F: CAGTTTTGGAAGCTTTCTGTGA
		R:GAACTCAACTGGGCCTTCTG

Supplementary Table 1. Primer sequences used for qRT-PCR.

Gene	Dharmacon Catalogue #
siControl	D-001210-01-20
si*ßCatenin*	M-003482-00
si*LGR4*	M-003673-03
si*LGR5*	M-005577-01
si*LRP5*	M-003844-02
si*LRP6*	M-003845-03
si*RSPO1*	M-018179-01
si*RSPO2*	M-017888-01
si*ZNRF3*	M-010747-02
si*RNF43*	M-007004-02

Supplementary Table 2. Catalog numbers of siRNA used in the study.

Gene	Sequence (5’-3’)
rspo2	GCCGTCCAAATGCAGTTTCAAC
chordin	1: ACGTTCTGTCTCGTATAGTGAGCGT
	2: ACAGCATTTTTGTGGTTGTCCCGAA
bmp4	CAGCATTCGGTTACCAGGAATCATG
lrp6	CCCCGGCTTCTCCGCTCCGACCCCT
znrf3	AACATAATTTCCCAGTCCTCAGTGG
rspo2$^{\Delta TSP}$	CAGCCATCTGGGAAGGCAACAGAAA

Supplementary Table 3. Antisense Morpholino sequences for *X.laevis* analyses.

Gene	Target Sequence(5’-3’)
rspo2	TGACTCCATAGTATCCAGGA
noggin	CCTGGGACTTAGAATAGACC
chordin	CTGCTGGTGTCTTAGATTGG

Supplementary Table 4. Primer sequences for *X.tropicalis* gRNAs.

Supplementary methods

All exact P values in the supplementary figures are as follows. (Left to right of the graph) **Supplementary Figure 1**; (**h**), <0.0001, 0.0016, 0.0265; (**k**), 0.2422, 0.2236, 0.0003, 0.0019; (**l**), 0.9990, 0.2752; **Supplementary Figure 3**; (**g**), <0.0001, 0.0024; (**i**), <0.0001, 0.0415 **Supplementary Figure 4**; (**b**), 0.4906, 0.0012, <0.0001 (**d**), <0.0001, 0.0022, 0.0016; (**f**), 0.0056, 0.0248, 0.0328, 0.0124; **Supplementary Figure 6**; (**d**), <0.0001, 0.0004, <0.0001, 0.0002.
