# Supplementary material for: R-spondins are BMP receptor antagonists in Xenopus early embryonic development
Source: Nat Commun. 2020 Nov 4;11:5570. doi: 10.1038/s41467-020-19373-w (PMC7642414; doi:10.1038/s41467-020-19373-w)
Supplement: Supplementary file 3 — Reporting Summary [file 41467_2020_19373_MOESM3_ESM.pdf]

nature research

Corresponding author(s): Christof Niehrs

Last updated by author(s): Sep 28, 2020

Reporting Summary

Nature Research wishes to improve the reproducibility of the work that we publish. This form provides structure for consistency and transparency in reporting. For further information on Nature Research policies, see our Editorial Policies and the Editorial Policy Checklist.

Statistics

For all statistical analyses, confirm that the following items are present in the figure legend, table legend, main text, or Methods section.

n/a	Confirmed	
☐	☒	The exact sample size (*n*) for each experimental group/condition, given as a discrete number and unit of measurement
☐	☒	A statement on whether measurements were taken from distinct samples or whether the same sample was measured repeatedly
☐	☒	The statistical test(s) used AND whether they are one- or two-sided<br>*Only common tests should be described solely by name; describe more complex techniques in the Methods section.*
☒	☐	A description of all covariates tested
☒	☐	A description of any assumptions or corrections, such as tests of normality and adjustment for multiple comparisons
☐	☒	A full description of the statistical parameters including central tendency (e.g. means) or other basic estimates (e.g. regression coefficient) AND variation (e.g. standard deviation) or associated estimates of uncertainty (e.g. confidence intervals)
☐	☒	For null hypothesis testing, the test statistic (e.g. *F*, *t*, *r*) with confidence intervals, effect sizes, degrees of freedom and *P* value noted<br>*Give P values as exact values whenever suitable.*
☒	☐	For Bayesian analysis, information on the choice of priors and Markov chain Monte Carlo settings
☒	☐	For hierarchical and complex designs, identification of the appropriate level for tests and full reporting of outcomes
☐	☒	Estimates of effect sizes (e.g. Cohen's *d*, Pearson's *r*), indicating how they were calculated

Our web collection on statistics for biologists contains articles on many of the points above.

Software and code

Policy information about availability of computer code

Data collection	No software was used to collect the data in this study.
Data analysis	GraphPad Prism 7 software, Image J v1.51k software, Adobe Photoshop CS6 software

For manuscripts utilizing custom algorithms or software that are central to the research but not yet described in published literature, software must be made available to editors and reviewers. We strongly encourage code deposition in a community repository (e.g. GitHub). See the Nature Research guidelines for submitting code & software for further information.

Data

Policy information about availability of data

All manuscripts must include a data availability statement. This statement should provide the following information, where applicable:

- Accession codes, unique identifiers, or web links for publicly available datasets
- A list of figures that have associated raw data
- A description of any restrictions on data availability

NCBI Reference Sequence
Xenopus tropicalis chordin : https://www.ncbi.nlm.nih.gov/nuccore/NM_001142657.1
Xenopus tropicalis noggin : https://www.ncbi.nlm.nih.gov/nuccore/NM_001171898.1?report=GenBank

All data in this study are provided in the Source Data file.
Raw images for the immunofluorescence microscopy is available upon reasonable request to the corresponding author.

Field-specific reporting

Please select the one below that is the best fit for your research. If you are not sure, read the appropriate sections before making your selection.

☒ Life sciences ☐ Behavioural & social sciences ☐ Ecological, evolutionary & environmental sciences

For a reference copy of the document with all sections, see nature.com/documents/nr-reporting-summary-flat.pdf

Life sciences study design

All studies must disclose on these points even when the disclosure is negative.

Sample size	No sample-size calculation was performed. Sample size was chosen by following literatures in the field and sufficient to execute statistical analyses.
Data exclusions	No data were excluded from the analyses.
Replication	BMP reporter assays were repeated 2-4 times independently. Western blot analyses for BMP signaling were repeated 2-3 times independently. Cell surface binding assays were repeated 2-5 times independently. Immunofluorescence microscopy analyses were repeated 2-3 times independently. Xenopus phenotype analyses were repeated 2-3 times independently. Xenopus in situ hybridization were repeated 2-4 times independently. Xenopus qRT-PCR was performed 3 times independently. We confirmed that these experiments showed similar results which draw the same conclusion.
Randomization	We splitted cultured cells and fertilized Xenopus eggs equally and randomly allocated to each of experimental group.
Blinding	Scoring of Xenopus phenotypes was performed under blinding (H.L, C.S)<br>Western blotting, reporter assay, binding assay, qRT-PCR analysis, immunofluorescence analysis, Xenopus in situ hybridization were performed by the authors (H.L, C.S., R.S) without blinding during data collection and analysis. Because the result of these objective evaluations were not affected by the impression of investigators.

Reporting for specific materials, systems and methods

We require information from authors about some types of materials, experimental systems and methods used in many studies. Here, indicate whether each material, system or method listed is relevant to your study. If you are not sure if a list item applies to your research, read the appropriate section before selecting a response.

Materials & experimental systems

n/a	Involved in the study	
☐	☒	Antibodies
☐	☒	Eukaryotic cell lines
☒	☐	Palaeontology and archaeology
☐	☒	Animals and other organisms
☒	☐	Human research participants
☒	☐	Clinical data
☒	☐	Dual use research of concern

Methods

n/a	Involved in the study	
☒	☐	ChIP-seq
☒	☐	Flow cytometry
☒	☐	MRI-based neuroimaging

Antibodies

Antibodies used

Primary antibodies
1. anti-Phospho-Smad1: CST (supplier), #9516 (catalog number), clone 41D10 (clone name), Lot 9 (Lot number)
2. anti-Smad1: CST, #9743, Lot 4
3. anti-Smad1: Santa Cruz, sc-7965, clone A4
4. anti-LRP6 : CST, #2560, clone C5C7, Lot 11
5. anti-Transferin receptor: CST, #13113S, clone D7G9X, Lot 1
6. anti-GAPDH : CST, #2118S, clone 14C10, Lot 10
7. anti-LAMP1 : abcam, #ab24170, clone ab25744
8. anti-beta-Catenin : BD, #610154
9. anti-DVL3 : CST, #3218S, Lot 2
10. anti-ROR2 : Santa Cruz, #sc374174, clone H-1, Lot A2412
11. anti-HA : Roche, #11867423001, clone 3F10
12. anti-Flag : Sigma, #3156, clone M2, Lot SLBN8915V
13. anti-GFP : ABIN, #100085, Lot 733301
14. anti-Myc : Invitrogen, clone 9E10, purified from hybridoma cells
15. anti-EEA1 : BD, #610457, clone 14/EEA1

	Secondary antibodies<br>1. Donkey anti-mouse Alexa 647 : Invitrogen, #A31571<br>2. Donkey anti-rat Alexa 488 : Invitrogen, #A21208<br>3. Donkey anti-rabbit 546 : Invitrogen, #A10036<br>4. Goat anti-mouse 488 : Invitrogen, #A11029<br>5. Goat anti-rabbit 546 : Invitrogen, #A11035
Validation	1. anti-Phospho-Smad1: Tested by manufactures in HeLa and NIH/3t3 using western blotting. Cited more than 100 times.<br>2. anti-Smad1 (CST): Tested by manufactures in HT1080, ACHN using western blotting. Cited more than 90 times.<br>3. anti-Smad1 (Santa Cruz): Tested by manufactures in NIH/3T3, Mv1 Lu cell lysates using western blotting.<br>4. anti-LRP6 : Tested by manufactures in HepG2, HeLa, Rat2 using western blotting. Cited more than 40 times. We tested with siRNA (Supplementary Fig. 1).<br>5. anti-Transferin receptor: Tested by manufactures in SNB75, ZR-75, HeLa, SNB19, HT-1080 cells using western blotting.<br>6. anti-GAPDH : Tested by manufactures in HeLa, NIH/3T3, C6, HUVEC, L929 cells using western blotting. Commonly used and cited more than 2000 times.<br>7. anti-LAMP1 : Tested by manufactures in Jurkat, A431, HEK293, MCF7 cells by western blotting. Cited more than 360 times.<br>8. anti-betaCatenin : Commonly used and cited more than 500 times. We validated using siRNA (Supplementary Fig. 1).<br>9. anti-DVL3 : Tested by manufactures using NIH/3T3, C6, A549, BAEC and COS cells by western blotting. Cited 20 times. We validated by siRNA (Supplementary Fig. 1).<br>10. anti-ROR2 : Tested by manufacturers using K-562, HeLa and U266 cells by western blotting. We validated by siRNA.<br>11. anti-EEA1 : Tested by manufacturers using immunofluorescent staining on human smooth muscle.<br>12. anti-HA : Tested by manufactures using immunoprecipitation of an HA-tagged GFP protein with anti-HA antibody.<br>13. anti-Flag : Commonly used and cited more than 3000 times. We validated by western blotting using Flag-tagged RSPO2 conditioned media comparing control conditioned media.<br>14. anti-GFP : Tested by manufacturers using mouse brain tissues for immunofluorescence and GFP protein in CHO cells for western blotting. Cited more than 90 times.<br>15. anti-Myc : Commonly used antibody. Tested by manufactures using HEK293T-c-myc cells and recombinant c-myc tagged proteins. We validated by western blotting using Myc-tagged RSPO2 conditioned media comparing control conditioned media. Cited more than 150 times.

Eukaryotic cell lines

Policy information about cell lines

Cell line source(s)	HEK293T (ATCC), HEPG2 (ATCC), H1581(ATCC; Gift from Dr. R. Thomas), L cells (ATCC)
Authentication	None of the cell lines were authenticated.
Mycoplasma contamination	All the cells were tested and negative for mycoplasma contamination.
Commonly misidentified lines (See ICLAC register)	No commonly misidentified lines used in this study.

Animals and other organisms

Policy information about studies involving animals; ARRIVE guidelines recommended for reporting animal research

Laboratory animals	Adults female Xenopus laevis and Xenopus tropicalis were used to obtain eggs. Adults male Xenopus laevis and Xenopus tropicalis were used to obtain testis for in vitro fertilization.<br>Xenopus laevis/tropicalis embryos at stage 10-11 were analyzed for in situ hybridization. Xenopus laevis embryos at stage 9-11.5 were analyzed for immunostaining. Xenopus laevis embryos at stage 10 were analyzed for qRT-PCR and animal cap in situ hybridization. Xenopus laevis/tropicalis tadpoles at stage 28-32 were analyzed for phenotype analyses.<br>Sex of the Xenopus embryos is not distinguishable at these stages and unrelevant to the study, therefore not determined.
Wild animals	The study did not involve wild animals.
Field-collected samples	The study did not involve field-collected samples.
Ethics oversight	The state review board of Baden-Wurttemberg, Germany (Permit number 35-9185.81/G-141/18 (Regierungspräsidium Karlsruhe))

Note that full information on the approval of the study protocol must also be provided in the manuscript.
